# Deep learning identified glioblastoma subtypes based on internal genomic expression ranks

**DOI:** 10.1186/s12885-022-09191-2

**Published:** 2022-01-20

**Authors:** Xing-gang Mao, Xiao-yan Xue, Ling Wang, Wei Lin, Xiang Zhang

**Affiliations:** 1 Department of Neurosurgery, Xijing Hospital, Fourth Military Medical University, Xi’an, Shaanxi Province People’s Republic of China; 2 Department of Pharmacology, School of Pharmacy, Fourth Military Medical University, Xi’an, Shaanxi Province People’s Republic of China; 3 State Key Laboratory for Manufacturing System Engineering, School of Mechanical Engineering, Xi’an Jiaotong University, Xi’an, 710054 China

**Keywords:** Deep neural network, Proneural, Neural, Classical, Mesenchymal, Machine learning, Molecular subtype, Glioma, Artificial intelligence, Support vector machines

## Abstract

**Background:**

Glioblastoma (GBM) can be divided into subtypes according to their genomic features, including Proneural (PN), Neural (NE), Classical (CL) and Mesenchymal (ME). However, it is a difficult task to unify various genomic expression profiles which were standardized with various procedures from different studies and to manually classify a given GBM sample into a subtype.

**Methods:**

An algorithm was developed to unify the genomic profiles of GBM samples into a standardized normal distribution (SND), based on their internal expression ranks. Deep neural networks (DNN) and convolutional DNN (CDNN) models were trained on original and SND data. In addition, expanded SND data by combining various The Cancer Genome Atlas (TCGA) datasets were used to improve the robustness and generalization capacity of the CDNN models.

**Results:**

The SND data kept unimodal distribution similar to their original data, and also kept the internal expression ranks of all genes for each sample. CDNN models trained on the SND data showed significantly higher accuracy compared to DNN and CDNN models trained on primary expression data. Interestingly, the CDNN models classified the NE subtype with the lowest accuracy in the GBM datasets, expanded datasets and in IDH wide type GBMs, consistent with the recent studies that NE subtype should be excluded. Furthermore, the CDNN models also recognized independent GBM datasets, even with small set of genomic expressions.

**Conclusions:**

The GBM expression profiles can be transformed into unified SND data, which can be used to train CDNN models with high accuracy and generalization capacity. These models suggested NE subtype may be not compatible with the 4 subtypes classification system.

**Supplementary Information:**

The online version contains supplementary material available at 10.1186/s12885-022-09191-2.

## Background

Glioblastoma (GBM) is one of the most lethal tumors affecting human, which is the most common primary malignant tumor in brain [1]. Despite advanced therapeutic techniques, the medial survival of GBM patients is only about 15 months after combined treatment of radio- and chemo-therapy after surgical resection of the tumor. The lack of effective treatment prompted investigation of the pathogenesis of GBM, especially by high-throughput molecular studies such as mRNA, miRNA, proteins, et al. [2–4]. Along with the progression of bio-techniques, the cost of tumor genome sequencing is becoming lower, which might be routine examinations for GBM in the future. Importantly, it is more and more widely recognized that high grade gliomas (HGGs) should be classified by molecular signatures rather than traditional WHO grades to more accurately reflect the therapeutic effects and clinical characteristics of HGGs [5, 6]. According to the molecular signature, GBM can be classified into 4 subtypes: Proneural (PN), Neural (NL), Classical (CL), and Mesenchymal (ME) [4], or into 3 subtypes by previous [7] and recent studies [8]. The classification is mainly based on clustering algorithms such as consensus average linkage hierarchical clustering. Normally, a cohort of genes were used to determine the subtype of a single sample. However, because of the complicated unification procedure of the gene expressions from different gene-chip platforms and research groups, for a given sample, it is still difficult to tell what kind of subtype it is.

Deep learning has great potential to deal with complicated biological data, and has been used to recognize genetic, histopathological, and radiographic features of GBM or low-grade glioma [9–14]. Particularly, deep learning showed values to predict molecular subtypes of low and high grade gliomas [15, 16], and to differentiate between gliomas and other central nervous system diseases [17]. Here, we developed an algorithm to transform the genomic expression of a single sample into unified standardized normal distribution (SND) data (SND-data) based only on the internal relative gene expression ranks of the sample itself. The transformed SND of GBM samples have same set of values but differed from each other by the orders of the values. This technique is rational according to the principles of delta-delta Ct methods widely used for quantitative PCR (qPCR) [18, 19], which actually gives a more precise relative value for a gene. Next, we build convolutional deep neural network (CDNN) to classify samples based on the SND-data [20, 21]. Although the SND-data may loss some information, it is sufficient to classify their subtypes by the CDNN. Therefore, the procedure of rank information-based unification of the genomic data into SND-data kept the relative ranking information, while lose certain detailed quantitative relationships, which is trivial considering the relatively great fluctuations in high-throughput data. Interestingly, as a result, subtype classification of GBM with the SND-data resulted in comparable or better accuracies to their original data, which can be used as a feasible tool to classify single GBM samples. More importantly, this unified procedure gives potential approaches to distinguish other molecular or clinical features of GBM or other kind of tumors by deep learning techniques.

## Material and methods

### Data acquisition

**Fig. 1:**
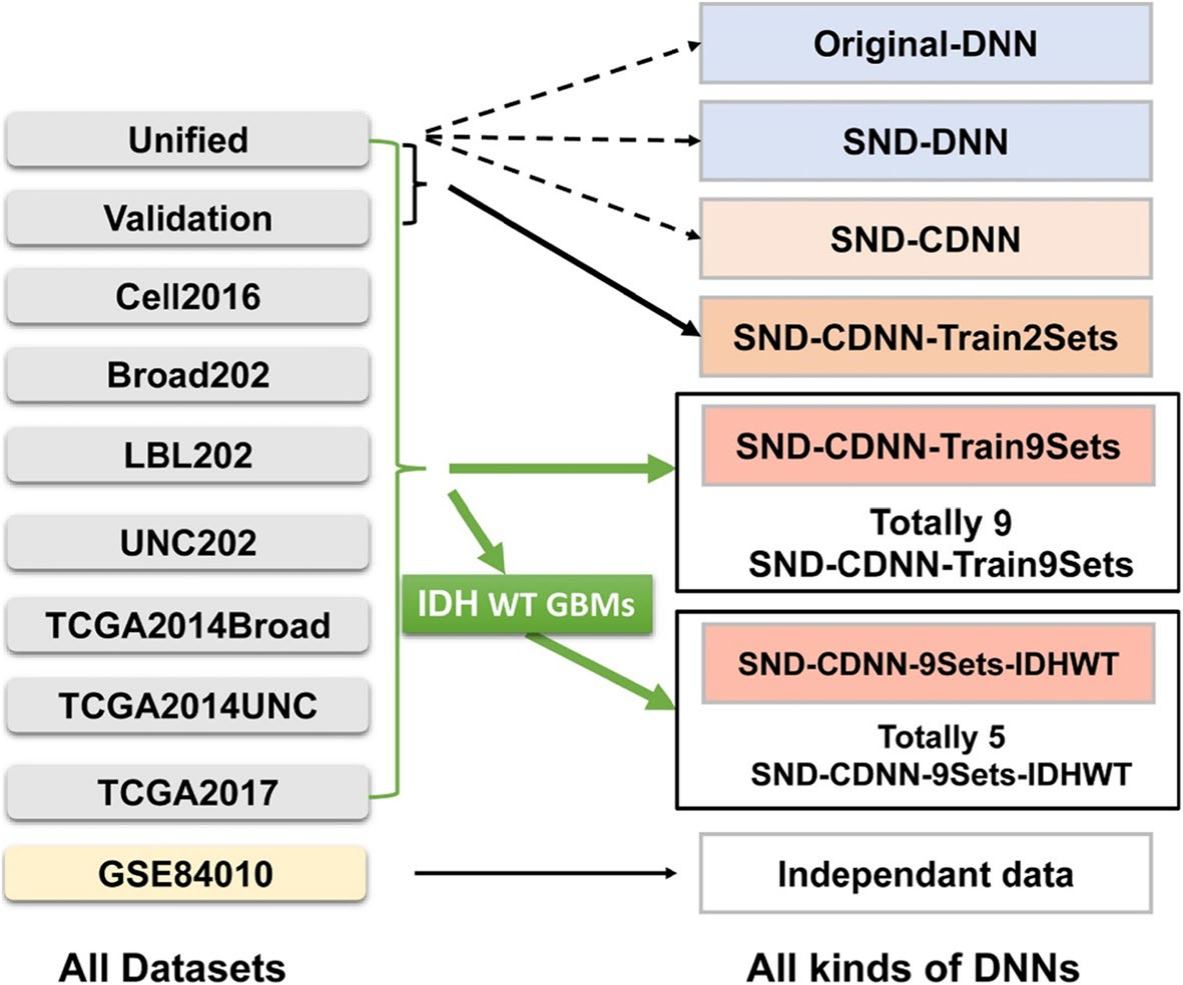
All datasets and trained NNs used in the present study. Totally 10 datasets, including 9 TCGA GBM datasets with different expression forms and 1 additional GBM dataset (GSE84010). Totally 2 types of NNs were used: DNN and CDNN. At last 6 kinds of NNs were trained, including original-DNNs trained in the unified dataset without data unification; SND-DNN trained in the unified dataset with data unification; SND-CDNN trained in the unified dataset with data unification; SND-CDNN-Train2Sets, which are trained in the combined unified and validation datasets with data unification; SND-CDNN-Train9Sets, which are trained in the combined 9 TCGA GBM datasets with data unification; SND-CDNN-9Sets-IDH-WT. which are trained in the IDH wide type GBM samples in the SND-Train9Sets with data unification

Totally 10 GBM datasets were used in the present study, among which 9 GBM datasets were used to train the neural networks (NNs, Fig. 1): 1 unified data and 1 validation data combining several datasets from Verhaak et al. (Unified and Validation data) [4], 1 standardized data from Ceccarelli et al. (Cell2016 data) [22], 3 original data provided in Verhaak et al. (Broad202, LBL202, UNC 202 data, which were processed in platforms of Affymetrix-HT-HG-U133A, Affymetrix HuEx GeneChip and Custom Agilent 244,000 feature Gene Expression Microarray, respectively) [4], and 3 TCGA datasets downloaded at different time points with different microarray platforms (TCGA2014Broad, TCGA2014UNC, TCGA2017 data). In addition, an independent GBM dataset downloaded from NCBI (GSE84010) [23], which was not included in the training dataset, was used as an additional validation dataset.

There are totally 4 kinds of datasets used for the DNN training: original Unified data (Original-Unified); Unified data transformed into “unified standardized normal distribution N(0, 1)” data (SND-Unified, see details below in Data Unification section); a combined dataset including Unified and Validation data transformed in to SND-data (SND-Train2Sets data); a combined data including all of the 9 training datasets (SND-Train9Sets). All of the datasets can be found in online materials.

### Deep learning training

Two kinds of NNs were developed: deep neural networks (DNNs) and convolutional deep neural networks (CDNNs). DNNs were composed by 4 layers, and CDNNs contained totally 5 layers: 2 convolutional layers, 1 subsampling layer, 1 density layer and 1 output layer. Large ranges of super-parameters were trained to get optimized DNNs with high accuracies, including number of iterations, epochs, dropout and momentum values, number of layers and number of nodes in each layer, activation functions in each layer, et al. For CDNNs, there are more super-parameters, including kernel size in the convolutional layers and stride values in the subsampling layer. For each kind of the above 4 training datasets, to train the DNNs, the dataset was shuffled and split into training (70%) and testing (30%) datasets. The trained DNNs was further validated in untrained datasets. For the SND-Train9Sets dataset, 10% of the randomly selected samples were first preserved as validation data (Train9Sets-ValidateData), then the remaining 90% samples (Train9Sets-TrainTestData) were further split into training (70%) and test (30%) datasets to train the DNNs.

Totally 6 kinds of NNs were trained in the present study (Fig. 1): 1. a DNN obtained by training the original Unifies dataset (Original-DNN); 2. a DNN obtained by training the SND-Unifies dataset (SND-DNN); 3. a CDNN obtained by training the SND-Unifies dataset (SND-CDNN); 4. a CDNN obtained by training the SND-Train2Stes (SND-CDNN-Train2Sets); 5. a cohort of CDNNs obtained by training the SND-Train9Sets (SND-CDNN-Train9Sets, and totally 9 SND-CDNN-Train9Sets models were obtained for statistical analysis); 6. a cohort of CDNNs obtained by training the IDH wide type GBM samples in the SND-Train9Sets (SND-CDNN-Train9Sets-IDH-WT, totally 5 SND-CDNN-Train9Sets-IDH-WT models were obtained for statistical analysis). All of the trained NNs were saved as files for further investigation and can be found in online materials.

### Gene expressions unified into SND-data

**Fig. 2:**
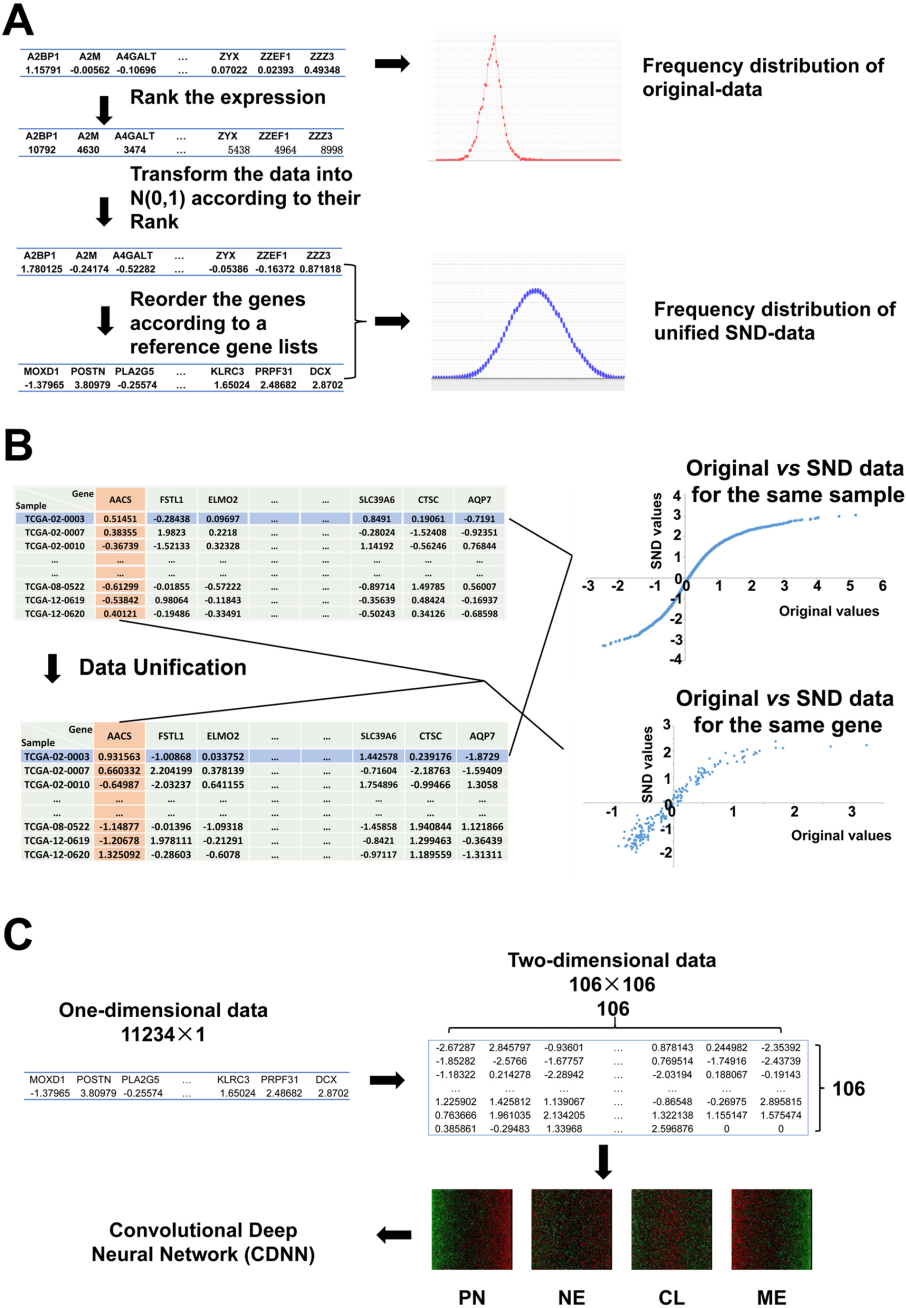
Data unification process of the GBM genomic expressions. **A** the expression data were first transformed into a unified N(0, 1) distribution based on the internal expression level ranks of all genes for each sample. Then the genes were ordered according to a fixed reference order. **B** the original-data and the SND-data kept certain correlations. **C** the one-dimensional expression data were arranged into a 2D-array data, which can be viewed as a “picture” and used in the CDNN training and testing

The widely used q-PCR is actually a method based on ranks of gene expression levels which determines the relative expression level of genes normalized to internal reference genes such as β-Actin or GAPDH. Based on these assumptions, the gene expressions of each sample were unified into SND-data by the following procedures (Fig. 2A):

1. Produce standardized normal distribution N(0, 1). Let the number of genes for each sample is *n*. We first generated a value array containing *n* elements obeying the N(0, 1) distribution, which is denoted as N(0, 1).
2. Rank the genes according to their internal expression levels. Order the gene expressions for each sample by their expression levels.
3. Transform the rank values of each gene into corresponding values with the same rank value in N(0, 1).

To ensure that different samples have comparability from each other, the genes were re-ordered according to a same fixed gene order (the reference gene list, RGL). In the present study, the RGL were defined by ordering the genes ascendingly according to their expression levels in the PN subtype in the unified dataset. If the gene in the RGL was not found in the dataset, then its value was set to a default value of 0. In addition, if the gene was not included in the RGL, then the value would be discarded.

### Implementation details

Our NNs implementation was based on the Deeplearning4J package, which is an open source, distributed deep-learning project in Java and Scala (Eclipse Deeplearning4j Development Team. Deeplearning4j: Open-source distributed deep learning for the JVM, Apache Software Foundation License 2.0. http://deeplearning4j.org).

### Analysis of classification accuracies for each GBM subtype

To investigate the detailed information of the classification results by the NNs, the classified results for each subtype were studied to calculate the accuracies for each subtype. For example, when we used the SND-CDNN-Train9Sets model trained on the Train9Sets-TrainTestData to classify the Train9Sets-ValidateData, samples in the Train9Sets-ValidateData labeled as PN subtype were classified by model as PN, NE, CL and ME for 52, 1, 0, and 4 times, respectively. Therefore, the accuracy for the PN subtype classification is 52/(52 + 1 + 0 + 4) = 91.23%. The classification accuracies for other subtypes were similarly calculated.

### Analysis of classification consistency between different groups

Classification of GBM samples based on their genomic expressions had been performed by different groups primarily based on data cluster analysis. The same TCGA datasets were then classified by different researchers with certain inconsistency. Here, we investigated the common GBM samples (totally 459 samples) classified by Brennan, C. W. et al. and Ceccarelli et al. (cell2013 and cell2016 data), respectively [2, 22]. Similarly, classification consistency for each subtype between the two groups was also calculated with the same procedures described above.

### Statistical analysis

Statistical analyses were performed using Student’s t-tests and one-way ANOVAs with least-squared-difference post-hoc tests, as appropriate. All *P*-values are 2-tailed, and *P* < 0.05 was considered statistically significant. Statistical analysis was performed with SPSS v.13.0.0.

## Results

### Deep Neural Network (DNN) classified GBM subtypes with high accuracy

The datasets used and DNN models investigated in the present work were elucidated in Fig. 1. We first used multilayer deep neural network (DNN) to classify GBM subtypes by using the TCGA dataset (Unified dataset) [4] containing 197 samples, which were classified into four subtypes: Proneural (PN), Neural (NL), Classical (CL), and Mesenchymal (ME). The unified dataset was split into training (70%) and testing datasets (30%) in random. Next, DNNs were constructed by using the gene expressions as input and the subtype of the sample as output (original-DNN). By investigating a large number of super parameters of the DNN, the best results can reach to accuracies at about 95.65% for the testing dataset. The architecture of the network was as follows: 4 layers with 1 input layer (11,234 nodes), 2 deep layers (760 and 120 nodes, respectively), and 1 output layer (4 nodes) (Supplementary Fig. S1, Table 1). The super parameters are: iterations = 5, number of epochs =2, learning rate = 0.005 (Table 1). We next used this network to classify other datasets that were not trained by the model. First, we tested the validation data containing about 260 samples [4]. As a result, our DNN model classified the validation data with an accuracy of 81.63%. Because the validation data is normalized with the similar process of the unified data which is used to train the DNN, we next tested whether the original-DNN have capacity to classify more generalized datasets. To do this, we used the original-DNN to classify the other 6 original TCGA GBM datasets which are not processed, including Broad202, LBL202, UNC202, TCGA2014Broad, TCGA2014UNC, and TCGA2017 datasets. As a result, the original-DNN classified these 6 datasets at accuracies only between 20.27% ~ 50.76% (30.93% ± 11.42%; Supplementary Table S1, Fig. 2), indicating the trained original-DNN may be over fitted or have difficulty to recognize datasets without normalization.

**Table 1:** Classification accuracies of the different networks to classify TCGA datasets into 4 subtypes: Proneural (PN), Neural (NL), Classical (CL), and Mesenchymal (ME)

Dataset/DNN model	DNN	SND-DNN	SND-CDNN	SND-CDNN-2Sets	SND-CDNN-9Sets
Broad202	20.27%	49.89%	52.56%	94.65%	94.92%
LBL202	28.43%	37.06%	60.91%	67.51%	95.94%
UNC202	28.43%	33.50%	59.39%	76.14%	94.42%
TCGA2014Broad	50.76%	57.87%	71.57%	82.74%	88.43%
TCGA2014UNC	20.98%	37.84%	50.78%	61.37%	91.07%
TCGA2017	36.72%	46.15%	57.07%	83.87%	76.55%
Mean	30.93	43.72	58.71	77.71%	90.22
SD	11.42%	9.25%	7.40%	12.03%	7.26%

### Data unification based on internal ranks of gene levels improved DNN performance

One of the major problems is that gene expressions in the above datasets were normalized with different approaches. Therefore, a key issue is to unify these datasets into a uniformed pattern. After data unification, gene expressions from different datasets should have the similar distribution. However, data unification is normally based on the distribution features of the whole dataset, making it difficult to transform and test single or a few samples. Therefore, we are trying to establish a simple unification method dependent on as few as additional data and investigate whether it can be used as input to classify the GBM samples by the DNN.

To unify the gene expressions, we first observed the data distribution of the gene expression. The frequency chart analysis revealed that the expressions are unimodally distributed and similar to normal distribution (Gaussian distribution, Fig. 2A). Therefore, the data unification process should keep this feature and the relative level ranks of all the genes. Notably, the widely used q-PCR is actually a method based on gene expression level ranks which gives the relative expression level of genes normalized to internal reference genes such as GAPDH or β-actin. Based on these assumptions, we transformed the gene expression data into a unified standardized normal distribution N(0, 1) (SND-data, see methods) (Fig. 2A).

After unification, the Original-data and the SND-data for each sample retained specific positive correlations similar to a sigmoid function (Fig. 2B). Interestingly, although the data is transformed independently for each sample, the data for each gene also kept specific correlations between the Original- and SND-data (Fig. 2B). These results demonstrated that our unification procedure kept key features of the dataset. The critical point of the unification procedure is that it transformed all of the expression data into unified form and can be feasibly used as input data for the DNN.

**Fig. 3:**
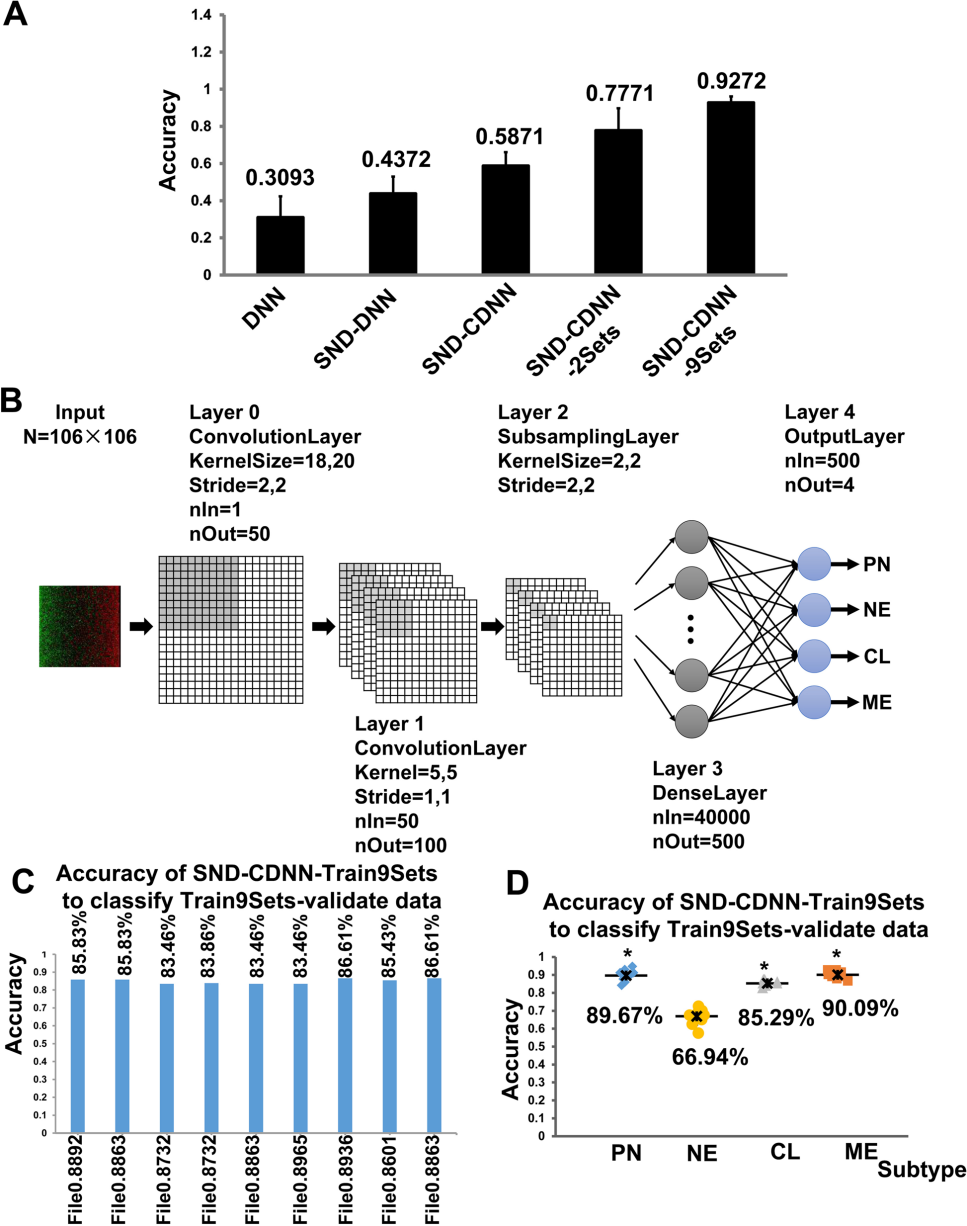
Architecture and classification accuracies of the CDNN. **A** Averaged classification accuracies of the DNN models on the 6 original TCGA datasets (Broad202, LBL202, UNC202, TCGA2014Broad, TCGA2014UNC, and TCGA2017). **B-D** The architecture and classification accuracies of the CDNN trained in the combined 9 TCGA GBM datasets (SND-CDNN-Train9Sets): **B** Architecture of the CDNN models; **C** Accuracies of a series of trained SND-CDNN-Train9Sets models on the Train9Set-validation data; **D** Subtype classification accuracies of the series of SND-CDNN-Train9Sets models on the Train9Set-validation data

Next, we used the normalized SND-data to train a DNN (SND-DNN). As a result, we got SND-DNNs with an accuracy of 97.10% at the testing dataset, slightly higher than that of DNN. Then we used the SND-DNN to classify the 6 original GBM datasets. The 6 GBM datasets were normalized into SND-data and then classified by the CDNN. As a result, the SND-CDNN classified these GBM datasets with accuracies between 33.5 ~ 57.87% (43.72% ± 9.25%), whose performance was improved when compared to the original-DNN (*p* < 0.01, paired t-test; Fig. 3A).

### Convolutional Deep Neural Network (CDNN) classified GBM subtypes with high accuracy and generalization capability

To further improve the classification accuracy and generalization capability, we next developed Convolutional Deep neural network (CDNN) to train the dataset. Because we have addressed that SND-data had better performance, we first normalized the data as described above, after which perform CDNN training (SND-CDNN). In fact, CDNNs obtained by training the original-data but not SND-data (original-CDNN) got an accuracy of more than 97% at the testing dataset, but these original-CDNN also have problems of generalization, which classified the other original-data with accuracies between 15.74 ~ 73.10%. Therefore, we next trained CDNNs by using the SND-data (SND-CDNN).

First, the data were normalized into SND-data, after which the one-dimensional gene expression SND-data were arranged into two-dimensional data array (2D-array) with square of the gene numbers as the length of the 2D-array. The missing data in the 2D-array was set into default value of 0 (Fig. 2C). Therefore, the 2D-array data can be viewed as pictures with different patterns and can be used as input data in a CDNN model (Fig. 2C). By using these 2D-array gene expression data as input, we trained the CDNN by optimizing the super parameters in a wide range of values, including the kernel size of the convolutional layer, number of layers, nodes numbers in each layer, et al. The detailed architecture of the SND-CDNN was shown in Fig. 3B, and the detailed super parameters are listed in Supplementary Table S2. At last, we got SND-CDNNs with accuracies more than 99% at the testing datasets, indicating SND-CDNN had better performances than multilayer DNNs. The SND-CDNN classified the validation dataset at an accuracy of 75.92%, smaller than that of SND-DNN. However, the SND-CDNN classified the other original datasets at accuracies between 50.78 ~ 71.57% (58.71% ± 7.40%), much better than that of SND-DNN (*p* < 0.01, paired t-test; Supplementary Table S1; Fig. 3A), indicating a higher generalization capacity of the SND-CDNN.

### Expended sample data improved the accuracy and generalization capacity of the CDNNs

We have addressed that SND-CDNN can significantly improve the accuracy and have high generalization capacity by introducing two critical techniques: 1. Normalizing the expression data into SND-data; 2. transforming the SND-data into 2-dimensional data as input for a CDNN. However, it should be noted that sample size is a critical factor for CDNN training and we only used a unified dataset counting 197 samples (actually 70% of the samples were used as training data) to train the CDNN. Therefore, we next added the validation data to the training datasets (designated as “Train2Sets”) and trained a new CDNN with the same super-parameters (SND-CDNN-Train2Sets). As a result, we obtained SND-CDNN-Train2Sets with accuracies more than 99% at the testing dataset. Interestingly, these SND-CDNN-Train2Sets classified the 6 original GBM datasets at accuracies of 61.37 ~ 83.87% (77.71% ± 12.03%; Table 1), which were significantly higher than that of the above SND-CDNN trained on only the unified dataset.

Next, we are trying to further expand the sample size of the training datasets. An important technique in deep learning is to expand the sample size by transforming the pictures such as scaling, rotation, et al. For the TCGA GBM dataset, many studies provided datasets processed with different procedures. Therefore, to expand the sample size, we combined totally 9 datasets, including the above unified data, validation data, the 6 original datasets and 1 dataset from Ceccarelli et al. [22], totally 2540 samples, which are designated as “Train9Sets”. First, 10% of the Train9Sets samples (Train9Sets-validate dataset) were randomly selected which were not used in the process of training the CDNN models and would serve as a validate dataset. Next, the remaining 90% Train9Sets samples were shuffled and split into training (70%) and testing samples (30%). By using the same super parameters of the above CDNN, we got SND-CDNN-Train9Sets models with accuracies of about 89% for the testing samples. The accuracies for the test datasets were relatively lower because these data comprised a wide range of expression patterns. Interestingly, a representative network model of SND-CDNN-Train9Sets classified the 6 original datasets with accuracies of 76.55 ~ 95.94% (90.22% ± 7.26%; Table 1, Fig. 3A; C), significantly better than the above SND-CDNN-Train2Sets.

**Fig. 4:**
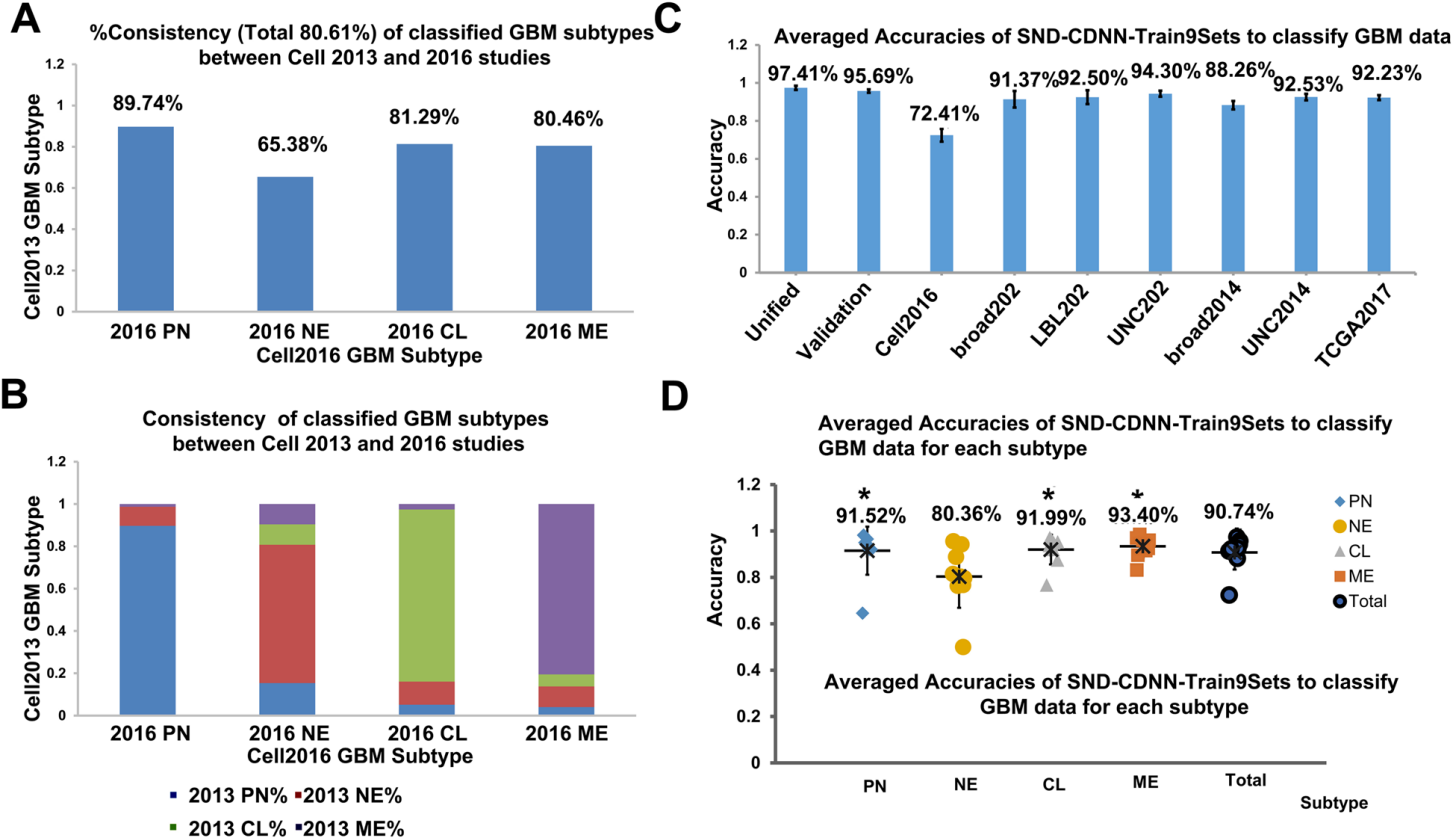
Consistencies of subtype classifications of TCGA GBM samples between different studies (**A-B**) and Averaged subtype classification accuracies of the SND-CDNN-Train9Sets models on each of the GBM dataset (**C-E**). * *p* < 0.01 compared to NE

To avoid biases derived from only one trained network model, we trained and got a series of SND-CDNN-Train9Sets models (totally 9 models). First, importantly, these SND-CDNN-Train9Sets classified the 10% Train9Sets-validate data, which are not included in the training process either as training or as testing data, at accuracies between 83% ~ 87% (Fig. 3D), indicating a high generalization capacity of the SND-CDNN-Train9Sets to recognize untrained data. This accuracy is acceptable, because the same GBM data classified by different research groups had a consistence of about 80%. For example, GBM subtype classification from studies of Brennan, C. W. et al. and Ceccarelli et al. (cell2013 and cell2016) resulted in a total consistency of about 80.61% to each other [2, 22] (Fig. 4A-B). Interestingly, the PN subtype showed the lowest consistency between the two results (Fig. 4A-B). In summary, the accuracy and generalization capacity of SND-CDNN can be improved substantially by expanding the GBM sample size with the same super parameters.

### SND-DCNN classified neural subtype with low accuracy

When we analyzed the detailed classification accuracies of the SND-CDNN-Train9Sets models to classify the 10% Train9Sets-validate data for each subtype, we noted that, interestingly, these models classified the NE subtype with an averaged accuracy of only 64.17% (55.00% ~ 70.00%), much lower than that of PN, CL and ME subtypes, which were classified at averaged accuracies of 91.23% (84.21% ~ 98.25%), 85.14% (82.43% ~ 86.49%) and 91.37% (89.16% ~ 92.77%), respectively (*p* < 0.001, ANOVA, Fig. 3D; Supplementary Table S3). Similar results were observed when these models were used to classify the whole Train9Sets data (Supplementary Fig. S2, Supplementary Table S4). Considering the recent report that the NE subtype may be non-tumor specific and should be excluded [8, 24], our identified CDNN actually implied the fact that the Neural subtype may be not compatible with the classification system.

To further confirm that CDNN classified NE subtype in a lower accuracy, we next used the SND-CDNN-Train9Sets models to classify all of the 9 GBM datasets. To get more reliable results, we analyzed the average values of all the models. We noted that the network classified most of the sets with accuracies more than 90% (7 of the 9 datasets were classified at accuracies more than 90%; Fig. 4C, Supplementary Table S5). Then we analyzed the detailed classification results for each subtype in these datasets. As a result, the SND-CDNN-Train9Sets classified NE subtype with an averaged accuracy of 80.36%, while they classified PE, CL and ME subtypes with averaged accuracies of 91.52, 91.99 and 93.40%, respectively (*p* < 0.05, ANOVA, Fig. 4C-D). These data suggested that SND-CDNN-Train9Sets models consistently classified the NE subtype with low accuracies, further supporting the concept that the NE subtype may not be compatible with the 4 subtypes classification system of GBM.

### SND-DCNN classified neural subtype with low accuracy in IDH-WT GBMs

**Fig. 5:**
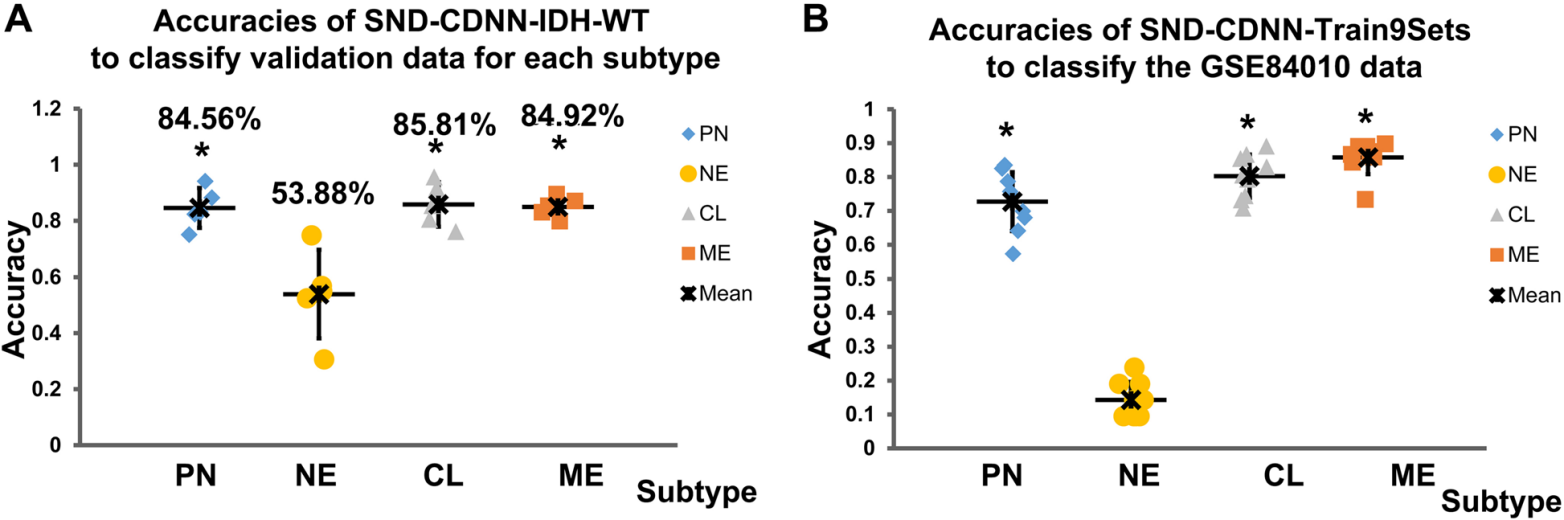
Averaged subtype classification accuracies of the SND-CDNN-Train9Sets-IDH-WT models on the IDH-WT GBM validation data (**A**), and Averaged subtype classification accuracies of the SND-CDNN-Train9Sets models on the independent GSE84010 GBM dataset (**B**). * *p* < 0.01 compared to NE

The new classification results which suggested to exclude the NE subtype, was obtained primarily from the IDH-WT GBMs [8]. Therefore, we next trained SND-CDNN-Train9Sets to classify the IDH-WT GBMs in the whole 9 sets data (SND-CDNN-Train9Sets-IDH-WT). Totally, 1210 IDH-WT samples were obtained. Similarly, 10% of the Train9Sets-IDH-WT samples (Train9Sets-validate-IDH-WT) were randomly selected as independent validation datasets not involved in training process. Next, the remaining 90% samples were shuffled and split into training (70%) and testing samples (30%). We obtained 5 SND-CDNN-9Sets-IDH-WT models, which classified the IDH-WT GBMs at accuracies between 78.66% ~ 83.90% (Fig. 5A; Supplementary Table S4). Interestingly, these models classified the PN, CL, and ME subtypes at highly comparable accuracies between 84.56% ~ 85.80%, while classified the NE subtype at accuracy of only about 53.88% (Fig. 5A; Supplementary Table S6), further confirming the above results that NE subtype may not be compatible with the 4 subtypes classification system of GBM, especially for IDH-WT GBMs.

### The CDNN also recognized gene profiles without full gene expressions

The above CDNN recognized a wide range of gene expression from TCGA data. We next tested whether the SND-CDNN-Train9Sets can classify dataset beyond the TCGA GBM data. In addition, the CDNN used whole gene expression as input, but in some situations, only a portion of the gene expressions were provided. Next, we used a Bevacizumab treated patients gene expression data (GSE84010) [23], which only contained 770 core genes for each sample. Here, the GSE84010 dataset used core genes for classification of the GBMs, which preserved the key gene expression features of each subtype. As a result, the 9 SND-CDNN-Train9Sets models classified the dataset at an averaged accuracy of 75.88% (70.65% ~ 79.34%). Strikingly, however, these CDNN models classified the Neural subtype with an averaged accuracy of only 14.29% (9.52% ~ 23.81%), while they classified the PN, CL and ME subtypes with averaged accuracies of 72.71% (57.28% ~ 83.50%), 80.22% (70.73% ~ 89.02%) and 75.88% (73.44% ~ 86.72%), respectively (*p* < 0.001, ANOVA, Fig. 5B, Supplementary Table S7). Although not ideal, the accuracies still reached to a high level comparable to the consistence (about 80%) between classification results of TCGA GBM samples from different groups (Fig. 4A-B). These results indicated that even for small set of gene expressions, the SND-CDNN-Train9Sets can recognize the essential expression features and classify the GBM samples with high accuracy.

## Discussion

In this study, we demonstrated the utility of deep learning to classify GBM subtypes. As a kind of malignant brain tumor without ideal treatment, more and more studies were performed to decipher the molecular and pathological characteristics of GBM. Molecular classification was considered to be more important than traditional pathological classifications in recent years. Based on genomic and clinical investigations, GBMs were classified into 4 subtypes with different features of genomic mutations, expression profiles and clinical characteristics, that is PN, NE, CL and ME subtypes. In addition, core gene signatures were identified to define each subtype. The initial classification of GBM subtypes were primarily based on genomic expression data clustering analysis. However, genomic based classification is mainly used for clustering analysis, and infeasible to classify single or a few samples.

Deep learning is useful to classify dataset, even for complicated data, such as genome expression profile, which is infeasible for human to recognize. Another critical issue in genomic data analysis is the normalization procedure of expression values. The normalization approach used in different research groups would have inconsistences, and would result in differences in the expression profiles. For example, the same TCGA samples classified by two groups have a total consistency of about 80% (cell2013, 2016, Fig. 4A, B). Therefore, it would be important to normalize the expression profiles into a unified pattern. Here we unified the expression data based on the internal sample gene level ranks, which would dependent only on the relative expression levels of the sample itself, but not on other samples. Apparently, this unification process would loss a considerable portion of detailed information. As elucidated in Fig. 2A, the data frequency curves are similar before and after the unification process, except that the unified data has a more regular rank among all genes. However, critical information was kept, such as the relative expression level ranks, and the distribution feature. This rank-based unification is reasonable, given that the commonly used qPCR actually calculates the relative levels of genes normalized to the internal reference genes. In addition, considering that high-throughput data are not as accuracy as traditional qPCR methods, the detailed information lost during the unification process is acceptable. Interestingly, our deep learning study revealed that the DNN performed on SND-Data is better than in Original-Data. Further training on more datasets confirmed the conclusion that the SND-Data can be used to classify the GBM samples with high accuracy. Notably, the correlation curves between SND-data and the original-data showed a “S” shape (Fig. 2), an important feature in many biological processes, and, notably, similar to the sigmoid activation function commonly used in DNNs.

Another technique we employed is to transform 1-dimeantinal expression data into 2-dimentional data like images. This process is used to take the advantages of the CDNN, which has been proved to have excellent performance to classify images. As a result, this process significantly improved the classification accuracy, implying this process is better for the deep learning based classification of GBM subtypes. Actually, when we observe the 2-dimentional data as images, we can distinguish the typical PN and ME samples (Fig. 2C).

Expanding the samples by transforming, rotating of the original images are important techniques to expand the training dataset commonly used in deep learning. Here, TCGA data were used and processed by many research groups, providing excellent expansions of the sample data. As revealed by the data of SND-CDNN-Train2Sets and SND-CDNN-Train9Sets, expanding sample size improved the performance of the SND-CDNN. Importantly, the SND-CDNNs trained with the expanding datasets showed excellent generalization capacity to recognize a wide range of datasets. It should be noted that the GBM subtype is classified according to data analysis, which is dependent on the algorithm process, and may result in certain inconsistencies (Fig. 4A-B). Therefore, the GBM samples are actually lack of definite labels like the common classification of labeled images in computer science. Given these considerations, the SND-CDNN-Train9Sets, which classified the GBM samples at accuracies near 90%, exhibited excellent capacity to classify GBM subtypes.

Another important finding is that, the SND-CDNN classified the NE subtype with low accuracy, a phenomenon observed in various situations, especially in the SND-CDNN-Train9Sets-IDH-WT results, which is consistent with the conclusions of recent study which suggested to classify GBM into 3 subtypes [8]. These results suggested that the CDNN have capacity to find incompatible labels of the input data, a capacity similar to the unsupervised classification by deep learning. Therefore, the present result is actually a combination of labeled classification and unsupervised classification. The results of the “unsupervised classification” portion are based on the GBM research background, and is implicated by deep learning classification results.

The present study was focused on deep neural networks, one of the widely used machine learning model. Therefore, it would be interesting to compare it with the other classical machine learning models, such as Support Vector Machine (SVM). We also studied and compared the SVM model to classify the GBM data, by using a LIBSVM program [25]. As a result, interestingly, SVM also classified the SND-data at higher accuracies than the original-data (Supplementary Fig. S3). When trained only on one GBM data (the unified GBM data), either on original or SND data, the SVM exhibited better performance to classify the 6 original datasets (Supplementary S3) than the SND-CDNN (Fig. 3A). However, when used larger datasets (Train2Sets and Train9Sets) as training data, SND-CDNNs exhibited better performances than SVM (77.71 ± 12.03% vs 60.89 ± 15.07, 92.72 ± 3.40% vs 89.79% ± 9.55%, Fig. 3A and Supplementary Fig. S3), indicating CDNNs have advantages in larger datasets with better generalization capacity. We further split the Train9Sets into training data (90%) and validation data (10%), which were used to train the SVM model (SVM- Train9Sets) and examine the prediction capacity. As a result, we got 6 SVM- Train9Sets models, which classified the validation data at accuracies of 85.43% ~ 88.58% (86.88% ± 1.33%, Fig. S4). Notably, SVM-Train9Sets also classified the NE subtype with the lowest accuracy (62.75% ± 9.05%, Supplementary Fig. S4). These results further supported our conclusions that, SND-data kept key information for classification and NE subtype is not compatible with the 4 subtypes classification.

Although exhibited better performance than the SVM models in larger datasets, the CDNN models still have some limitations. First, because the fundamental principles of the CDNN is not fully clarified, this model has little value to provide insights into the underlying biological processes, and therefore has poor interpretability when it comes to translational cancer genomics. Next, similarly, although the CDNN models had better performances than the DNN, it is difficult to demonstrate the exact meanings of the transformed two-dimensional data of the genomic profiles. Nevertheless, in practice, given that obtaining the genomic profiles of a given sample would need lower price and shorter time in the future, it would be acceptable to identify the subtype of a GBM sample based on its genomic profile. The present work provided potential ways to make this process more feasible. Specifically, the internal-rank based SND transformation provides a concise algorithm to unify the genomic data. In addition, because sample size is important for the training of deep learning models, accumulated data in the future would further improve the performance of the CDNN models.

## Conclusion

In conclusion, the present work established approaches to normalize and classify GBM samples based only on their internal ranks of the genome data. Several networks were trained on the internal rank data of the genomic profile to classify GBM subtypes with high performance. In addition, the CDNNs analysis suggested to exclude the NE subtype from the four GBM subtype classification system.

## Supplementary Information

**Additional file 1: Supplementary Table S1.** Super parameters for the DNN. **Supplementary Table S2.** Super parameters for the CDNN. **Supplementary Table S3.** Averaged Accuracies of SND-CDNN-Train9Sets to classify Train9Sets-validate data for each subtype. **Supplementary Table S4.** Averaged Accuracies of SND-CDNN-Train9Sets to classify the whole Train9Sets data for each subtype. **Supplementary Table S5.** Averaged Accuracies of SND-CDNN-Train9Sets to classify GBM datasets for each subtype. **Supplementary Table S6.** Accuracies of SND-CDNN-Train9Sets-IDH-WT to classify the corresponding 10% validation datasets for each subtype. **Supplementary Table S7.** Accuracies of SND-CDNN-Train9Sets to classify the GSE84010 dataset for each subtype. **Supplementary Fig. S1.** Deep neural network architecture of the DNN models. **Supplementary Fig. S2.** Averaged subtype classification accuracies of the SND-CDNN-Train9Sets models on the whole combined GBM dataset (Train9Sets data). * *p* < 0.01 compared to NE. **Supplementary Fig. S3.** Averaged classification accuracies of the SVM models on the 6 original TCGA datasets (Broad202, LBL202, UNC202, TCGA2014Broad, TCGA2014UNC, and TCGA2017). **Supplementary Fig. S4.** Averaged subtype classification accuracies of the SVM-Train9Sets models (trained on 90% Train9Sets data) on the validation dataset (10% Train9Sets data). * *p* < 0.01 compared to NE.

## Data Availability

GBM data sets, CDNN models and computer code used in the study can be found in Mendeley data: Mao, Xing-gang (2021), “CDNN”, Mendeley Data, V2, doi: 10.17632/jjgtktxht5.2. Public access to the data is open and can be found and downloaded from: https://data.mendeley.com/datasets/jjgtktxht5/2. This is a permanent link which are opened and can be accessed without accession number. The links to all of the datasets were as follows: 1_unified.txt: https://data.mendeley.com/public-files/datasets/jjgtktxht5/files/a0a4edf0-f537-41ad-ab88-2be09e93fc96/file_downloaded 2_Validation_CommonGenes.txt: https://data.mendeley.com/public-files/datasets/jjgtktxht5/files/fbe393e9-86e1-47d0-b095-663359f2e6ed/file_downloaded 3_cell2016 (GBMLGG_EB_RmDiffFullGenesRanRmDup).txt: https://data.mendeley.com/public-files/datasets/jjgtktxht5/files/4eb7e509-485e-4cfc-8ad4-704d5f6041d8/file_downloaded 4_Broad202.txt: https://data.mendeley.com/public-files/datasets/jjgtktxht5/files/1f4f552a-d13d-460c-aeb5-eaeceecacab3/file_downloaded 5_LBL202.txt: https://data.mendeley.com/public-files/datasets/jjgtktxht5/files/10e2bee0-3b30-4f33-8afc-9e48fd1e5cd9/file_downloaded 6_UNC202.txt: https://data.mendeley.com/public-files/datasets/jjgtktxht5/files/4b70e9ad-cece-4992-b198-121885155896/file_downloaded 7_TCGA_2014Broad (GBM__broad.mit.edu__ht_hg-u133a__gene.quantification__Jul-08-2014).txt: https://data.mendeley.com/public-files/datasets/jjgtktxht5/files/8559b8b1-d847-44bf-90da-70dd02d62ba0/file_downloaded 8_TCGA_2014UNC (GBM__unc.edu__agilentg4502a_07_2__gene.quantification__Jul-08-2014).txt: https://data.mendeley.com/public-files/datasets/jjgtktxht5/files/629d1598-6f1f-4cb7-a46d-bedd8edc895c/file_downloaded 9_TCGA2017 (GBMinColumn).txt: https://data.mendeley.com/public-files/datasets/jjgtktxht5/files/7b42f40d-6602-4445-a20d-00ea98e38f03/file_downloaded GSE84010_Patients Bevacizumab data.txt: https://data.mendeley.com/public-files/datasets/jjgtktxht5/files/8f4eeb9e-d786-4ccf-bb70-7ffce1381e03/file_downloaded

## Abbreviations

CDNN: Convolutional deep neural network
CL: Classical
DNN: Deep neural networks
GBM: Glioblastoma
ME: Mesenchymal
NE: Neural
NNs: Neural networks
PN: Proneural
SND: Standardized normal distribution
SVM: Support Vector Machine
TCGA: The cancer genome atlas
HGGs: High grade gliomas
qPCR: Quantitative PCR
RGL: The reference gene list
