# Supplementary material for: Deep learning identified glioblastoma subtypes based on internal genomic expression ranks
Source: BMC Cancer. 2022 Jan 20;22:86. doi: 10.1186/s12885-022-09191-2 (PMC8780813; doi:10.1186/s12885-022-09191-2)
Supplement: Supplementary file 1 — Additional file 1: Supplementary Table S1. Super parameters for the DNN. Supplementary Table S2. Super parameters for the CDNN. Supplementary Table S3. Averaged Accuracies of SND-CDNN-Train9Sets to classify Train9Sets-validate data for each subtype. Supplementary Table S4. Averaged Accuracies of SND-CDNN-Train9Sets to classify the whole Train9Sets data for each subtype. Supplementary Table S5. Averaged Accuracies of SND-CDNN-Train9Sets to classify GBM datasets for each subtype. Supplementary Table S6. Accuracies of SND-CDNN-Train9Sets-IDH-WT to classify the corresponding 10% validation datasets for each subtype. Supplementary Table S7. Accuracies of SND-CDNN-Train9Sets to classify the GSE84010 dataset for each subtype. Supplementary Fig. S1. Deep neural network architecture of the DNN models. Supplementary Fig. S2. Averaged subtype classification accuracies of the SND-CDNN-Train9Sets models on the whole combined GBM dataset (Train9Sets data). * p < 0.01 compared to NE. Supplementary Fig. S3. Averaged classification accuracies of the SVM models on the 6 original TCGA datasets (Broad202, LBL202, UNC202, TCGA2014Broad, TCGA2014UNC, and TCGA2017). Supplementary Fig. S4. Averaged subtype classification accuracies of the SVM-Train9Sets models (trained on 90% Train9Sets data) on the validation dataset (10% Train9Sets data). * p < 0.01 compared to NE. [file 12885_2022_9191_MOESM1_ESM.docx]

Supplementary Table S1. super parameters for the DNN

| Super parameters | Values |
| --- | --- |
| iterations | 5 |
| number of epochs | 2 |
| dropout | 0.95 |
| momentum | 0.95 |
| backpropagation | true |
| Number of layers | 3 |
| Activation function in all layers | ReLu, ReLu, sigmoid |
| Number of nodes in each layer | 11234, 760, 120, 4 |

Supplementary Table S2. super parameters for the CDNN

| Super parameters | Values |
| --- | --- |
| iterations | 5 |
| number of epochs | 2 |
| dropout | 0.95 |
| momentum | 0.95 |
| backpropagation | true |
| Number of layers | 5 |
| Number of Con layers | 2 |
| Number of Sub layers | 1 |
| Number of density layers (include output layer) | 2 |
| Kernel size (in Con and Sub layers) | (18, 20), (5, 5), (2, 2) |
| Stride (in Con and Sub layers) | (2, 2), (1, 1), (2, 2) |
| Activation function in all layers | Identity, Identity, sigmoid, ReLu, softmax |

Con layers: Convolutional layers; Sub layers: subsampling layers; ReLu: Rectified Linear Units

Supplementary Table S3. Averaged Accuracies of SND-CDNN-Train9Sets to classify Train9Sets-validate data for each subtype.

| **Data**  **subtype** | cnn0.8892 | cnn0.8863 | cnn0.8732 | cnn0.8732 | cnn0.8863 | cnn0.8965 | cnn0.8936 | cnn0.8601 | cnn0.8863 | **Mean** | **SD** |
| --- | --- | --- | --- | --- | --- | --- | --- | --- | --- | --- | --- |
| PN | 0.9123 | 0.8947 | 0.8772 | 0.8947 | 0.9123 | 0.8421 | 0.9298 | 0.8596 | 0.9474 | **0.8967** | **0.0333** |
| NE | 0.7250 | 0.6750 | 0.6250 | 0.7000 | 0.5750 | 0.6500 | 0.6750 | 0.7000 | 0.7000 | **0.6694** | **0.0464** |
| CL | 0.8514 | 0.8784 | 0.8784 | 0.8243 | 0.8378 | 0.8243 | 0.8514 | 0.8649 | 0.8649 | **0.8529** | **0.0208** |
| ME | 0.8916 | 0.9036 | 0.8675 | 0.8795 | 0.9036 | 0.9277 | 0.9277 | 0.9157 | 0.8916 | **0.9009** | **0.0207** |
| Total | 0.8583 | 0.8583 | 0.8346 | 0.8386 | 0.8346 | 0.8346 | 0.8661 | 0.8543 | 0.8661 | **0.8495** | **0.0137** |

Supplementary Table S4. Averaged Accuracies of SND-CDNN-Train9Sets to classify the whole Train9Sets data for each subtype.

| **Data**  **subtype** | cnn0.8892 | cnn0.8863 | cnn0.8732 | cnn0.8732 | cnn0.8863 | cnn0.8965 | cnn0.8936 | cnn0.8601 | cnn0.8863 | **Mean** | **SD** |
| --- | --- | --- | --- | --- | --- | --- | --- | --- | --- | --- | --- |
| PN | 0.9441 | 0.9288 | 0.9119 | 0.9356 | 0.9356 | 0.9525 | 0.9644 | 0.8729 | 0.9576 | **0.9337** | **0.0278** |
| NE | 0.8614 | 0.8171 | 0.7552 | 0.8496 | 0.8142 | 0.7906 | 0.8201 | 0.7640 | 0.8437 | **0.8128** | **0.0370** |
| CL | 0.9217 | 0.9295 | 0.9478 | 0.9138 | 0.9008 | 0.9243 | 0.9138 | 0.9204 | 0.9347 | **0.9230** | **0.0135** |
| ME | 0.9065 | 0.9325 | 0.9089 | 0.9148 | 0.9444 | 0.9314 | 0.9290 | 0.9491 | 0.9231 | **0.9266** | **0.0148** |
| Total | 0.9138 | 0.9154 | 0.9008 | 0.9106 | 0.9118 | 0.9154 | 0.9181 | 0.8980 | 0.9240 | **0.9120** | **0.0081** |

Supplementary Table S5. Averaged Accuracies of SND-CDNN-Train9Sets to classify GBM datasets for each subtype.

| **Data**  **subtype** | unified | validation | TCGA2017 | broad202 | lBL202 | UNC202 | broad2014 | unc2014 | cell2016 | **mean** | **var** |
| --- | --- | --- | --- | --- | --- | --- | --- | --- | --- | --- | --- |
| PN | 0.9821 | 0.9657 | 0.6461 | 0.9206 | 0.9444 | 0.9504 | 0.9200 | 0.9808 | 0.9264 | **0.9152** | **0.1037** |
| NE | 0.9427 | 0.9556 | 0.5000 | 0.8029 | 0.7634 | 0.8889 | 0.7969 | 0.7672 | 0.8148 | **0.8036** | **0.1346** |
| CL | 0.9706 | 0.9436 | 0.7669 | 0.9203 | 0.9644 | 0.9371 | 0.8743 | 0.9490 | 0.9526 | **0.9199** | **0.0640** |
| ME | 0.9864 | 0.9610 | 0.8323 | 0.9610 | 0.9571 | 0.9708 | 0.8958 | 0.9157 | 0.9259 | **0.9340** | **0.0478** |
| Total | 0.9741 | 0.9569 | 0.7241 | 0.9137 | 0.9250 | 0.9430 | 0.8826 | 0.9253 | 0.9223 | **0.9074** | **0.0735** |

Supplementary Table S6. Accuracies of SND-CDNN-Train9Sets-IDH-WT to classify the corresponding 10% validation datasets for each subtype.

| **CDNN**  **subtype** | unified | validation | TCGA2017 | broad202 | lBL202 | **mean** | **var** |
| --- | --- | --- | --- | --- | --- | --- | --- |
| PN | 0.8824 | 0.8309 | 0.8235 | 0.9412 | 0.7500 | 0.8824 | 0.0713 |
| NE | 0.5485 | 0.5243 | 0.7476 | 0.3058 | 0.5680 | 0.5485 | 0.1573 |
| CL | 0.7605 | 0.9557 | 0.8537 | 0.8049 | 0.9157 | 0.7605 | 0.0794 |
| ME | 0.8531 | 0.7977 | 0.8702 | 0.8950 | 0.8302 | 0.8531 | 0.0373 |
| Total | 0.7866 | 0.8142 | 0.8390 | 0.7922 | 0.8045 | 0.7866 | 0.0207 |

Supplementary Table S7. Accuracies of SND-CDNN-Train9Sets to classify the GSE84010 dataset for each subtype.

| **Data**  **subtype** | cnn0.8892 | cnn0.8863 | cnn0.8732 | cnn0.8732 | cnn0.8863 | cnn0.8965 | cnn0.8936 | cnn0.8601 | cnn0.8863 | **Mean** | **SD** |
| --- | --- | --- | --- | --- | --- | --- | --- | --- | --- | --- | --- |
| PN | 0.6796 | 0.5728 | 0.7864 | 0.6408 | 0.8252 | 0.8350 | 0.7573 | 0.7476 | 0.6990 | **0.7271** | **0.0869** |
| NE | 0.1429 | 0.1905 | 0.0952 | 0.0952 | 0.0952 | 0.0952 | 0.1429 | 0.2381 | 0.1905 | **0.1429** | **0.0532** |
| CL | 0.8293 | 0.7439 | 0.7073 | 0.8537 | 0.7927 | 0.8049 | 0.7317 | 0.8902 | 0.8659 | **0.8022** | **0.0639** |
| ME | 0.8906 | 0.8750 | 0.8984 | 0.8906 | 0.8438 | 0.7344 | 0.8672 | 0.8594 | 0.8594 | **0.8576** | **0.0495** |
| Total | 0.7635 | 0.7066 | 0.7665 | 0.7545 | 0.7784 | 0.7425 | 0.7545 | 0.7934 | 0.7695 | **0.7588** | **0.0245** |

Supplementary Figures and legends


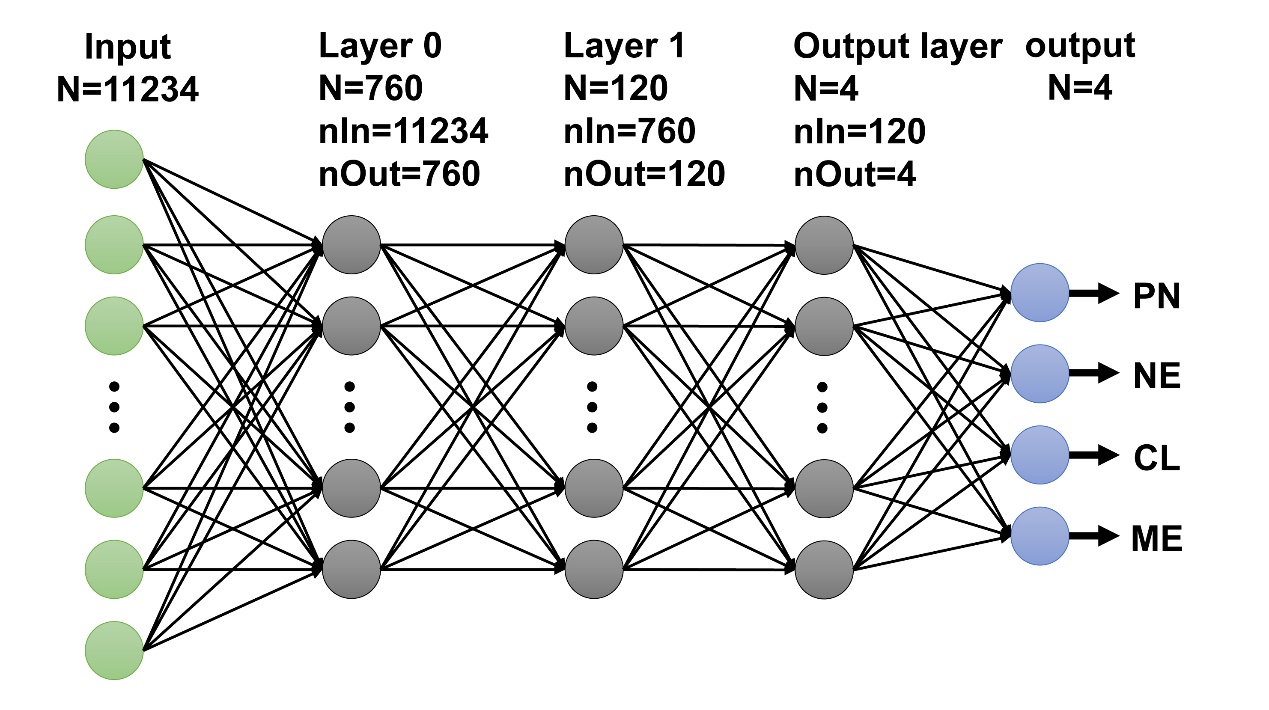


Supplementary Fig. S1. Deep neural network architecture of the DNN models.


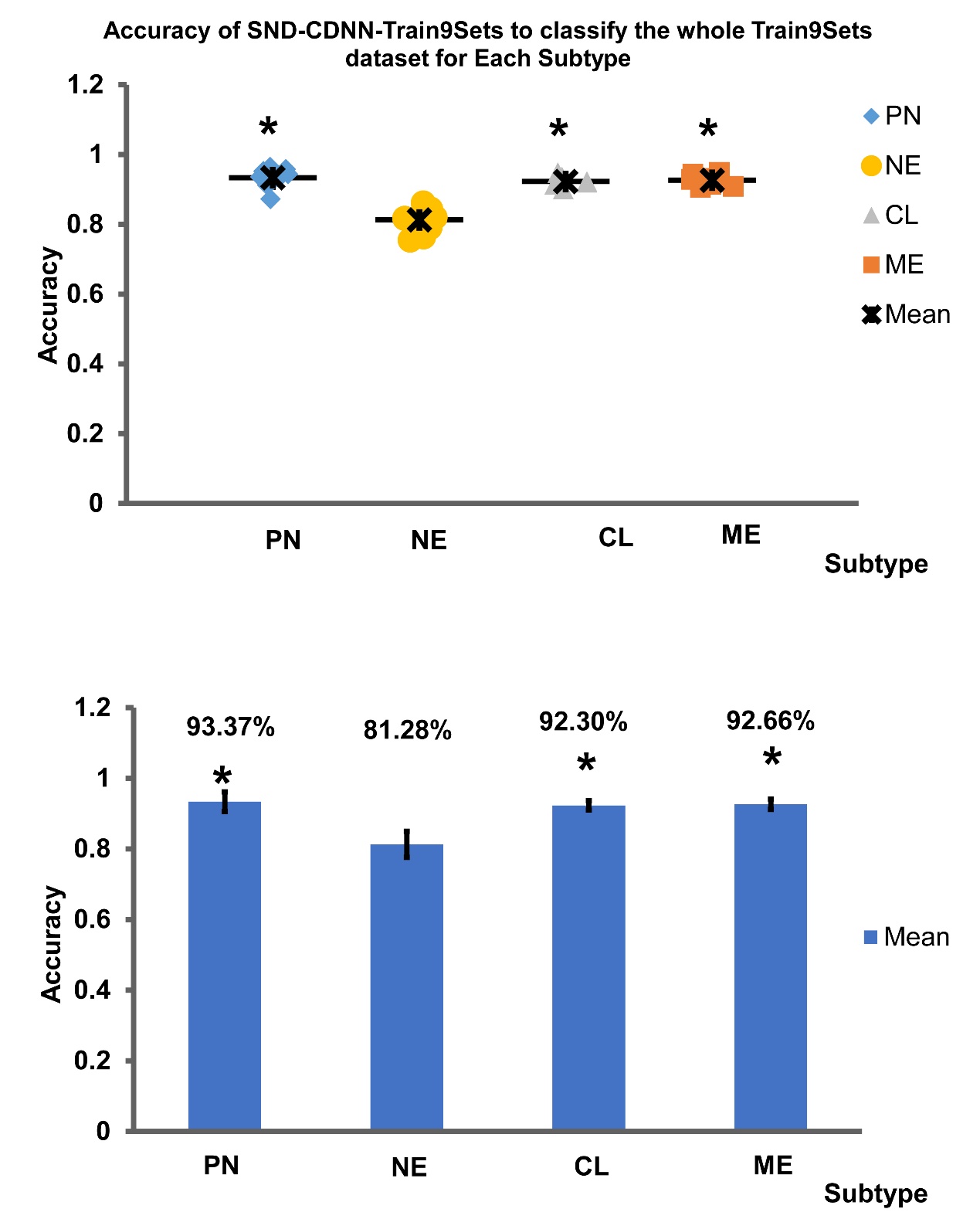


Supplementary Fig. S2. Averaged subtype classification accuracies of the SND-CDNN-Train9Sets models on the whole combined GBM dataset (Train9Sets data). * p<0.01 compared to NE.


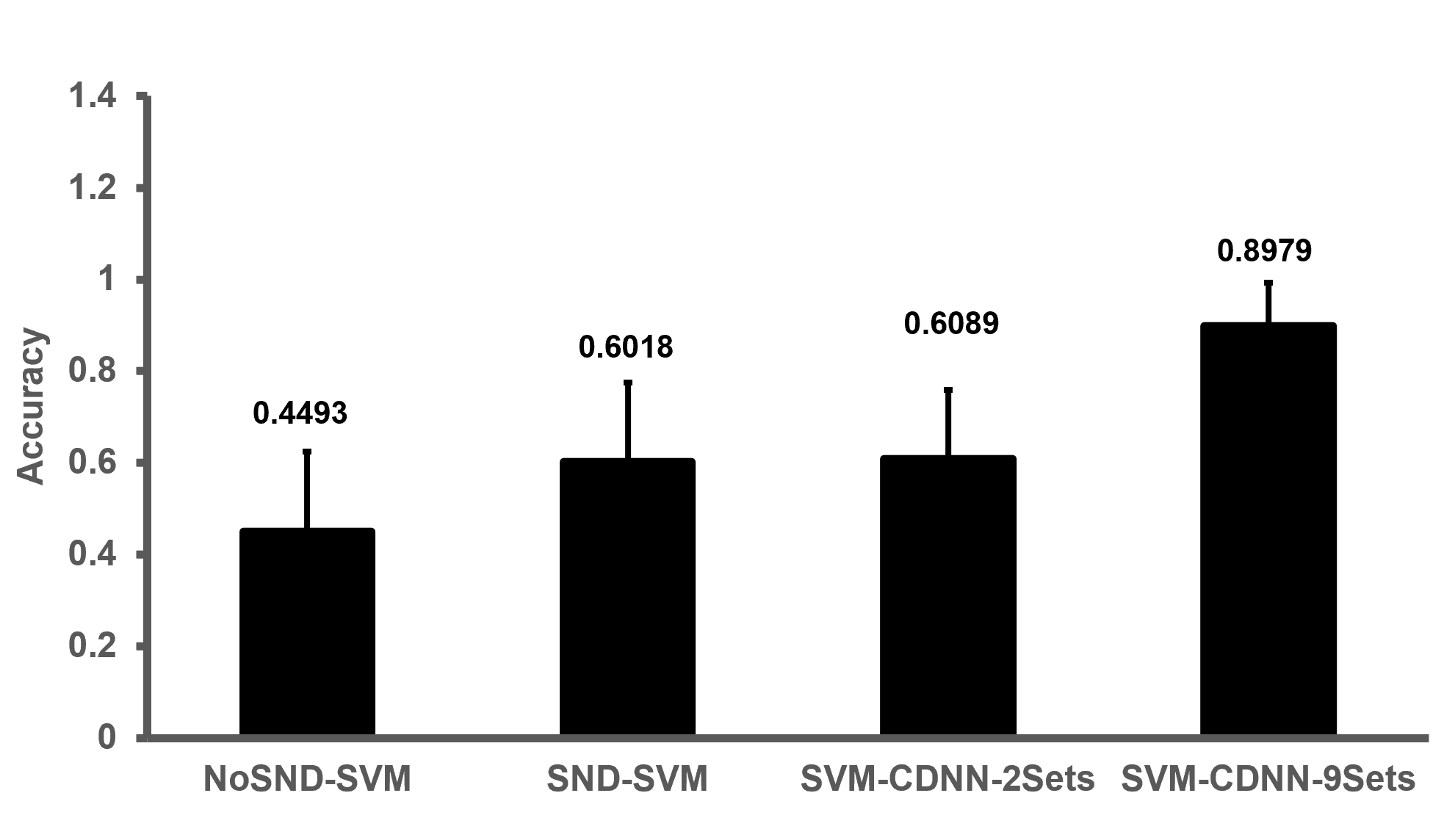


Supplementary Fig. S3. Averaged classification accuracies of the SVM models on the 6 original TCGA datasets (Broad202, LBL202, UNC202, TCGA2014Broad, TCGA2014UNC, and TCGA2017).


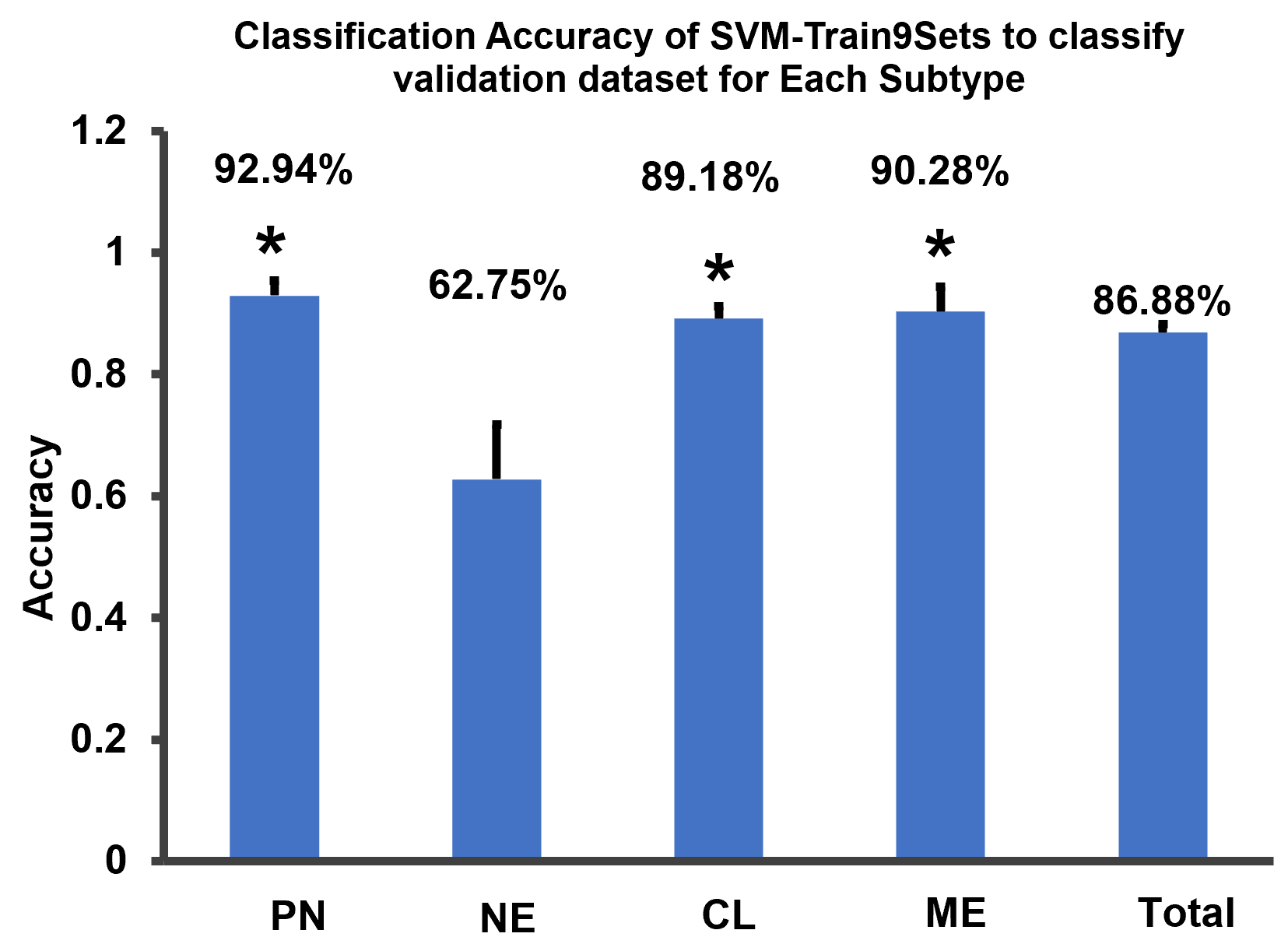


Supplementary Fig. S4. Averaged subtype classification accuracies of the SVM-Train9Sets models (trained on 90% Train9Sets data) on the validation dataset (10% Train9Sets data). * p<0.01 compared to NE.
